# A Thematic Analysis of Multiple Pathways Between Nature Engagement Activities and Well-Being

**DOI:** 10.3389/fpsyg.2021.580992

**Published:** 2021-03-26

**Authors:** Anam Iqbal, Warren Mansell

**Affiliations:** Faculty of Biology, Medicine and Health, School of Health Sciences, University of Manchester, Manchester, United Kingdom

**Keywords:** nature engagement, eco psychology, mental health, environment, well-being, mechanisms of change

## Abstract

Research studies have identified various different mechanisms in the effects of nature engagement on well-being and mental health. However, rarely are multiple pathways examined in the same study and little use has been made of first-hand, experiential accounts through interviews. Therefore, a semi-structured interview was conducted with seven female students who identified the role of nature engagement in their well-being and mental health. After applying thematic analysis, 11 themes were extracted from the data set, which were: “enjoying the different sensory input,” “calm nature facilitates a calm mood,” “enhancing decision making and forming action plans,” “enhancing efficiency and productivity,” “alleviating pressure from society's expectations regarding education,” “formation of community relations,” “nature puts things into perspective,” “liking the contrast from the urban environment,” “feel freedom,” “coping mechanism,” and “anxious if prevented or restricted.” The results indicate complementary mechanisms for how nature-related activities benefit mental health and well-being that may occupy different levels of experience within a hierarchical framework informed by perceptual control theory.

## Introduction

It has become increasingly recognized that nature engagement activities provide a convenient, effective, community-wide means to support mental health and well-being, especially during childhood (Bragg and Atkins, 2016). A recent Delphi study of 19 experts from seven countries collated the diverse range of nature engagement interventions and their potential well-being and health outcomes (Shanahan et al., 2019). They encompass ways to change people's surroundings (e.g., parks, workplaces), and widespread types of activities (e.g., adventure sports, wilderness play, outdoor therapy). Well-being has been conceptualized from a range of perspectives, including subjective well-being (Diener et al., 1999), social well-being (Larson, 1993), and psychological well-being (Ryff and Keyes, 1995). Each of these perspectives on well-being presents multifaceted constructs. For example, subjective well-being includes the phenomena of pleasant and unpleasant affect, life satisfaction and satisfaction in specific domains (e.g., work, family, health) (Diener et al., 1999). Social well-being combines social adjustment (including relationship satisfaction) and social support from one's network of contacts (Larson, 1993). Psychological well-being is also a multifaceted construct (Ryff and Keyes, 1995) and includes autonomy, environmental mastery, personal growth, positive relations, purpose in life and self-acceptance; these elements are generally considered to hold value (also known as eudaimonic well-being) but not necessarily pleasure (hedonic well-being). A wide range of inter-related mechanisms through which nature engagement may have its effects on well-being have been put forward, but rarely are they studied together, nor have the mechanisms been analyzed in the context of different perspectives on well-being. The current study aimed to discover the first-hand experience of potential mechanisms by interviewing seven people who identified as utilizing nature engagement for their well-being and mental health. We did not impose a specific well-being perspective but allowed participants to generate the facets of well-being that were meaningful to them.

A number of theories are grounded in evolution theory, pointing to the natural affinity of humans for natural environments, animals and plants—commonly described as the biophilia hypothesis (Wilson, 1984; Kellert and Wilson, 1995; Ewert et al., 2014). The sensory input from nature itself may reduce anxiety, owing to an innate drive to seek sensory experiences that eminate from nature (Ewert et al., 2014). These experiential properties may allow the individual to meet fundamental needs (Landon et al., 2020) such as autonomy, competence and relatedness, as specified by Self-Determination Theory (SDT; Deci and Ryan, 1985). The visual qualities of natural scenes are the focus of Stress Reduction Theory (Ulrich, 1983; Ulrich et al., 1991) that specifies the “preferenda”—the aesthetic qualities of natural scenes—that entail specific affective, cognitive and physiological states. These include the textures, depth cues, level of complexity, and configurations within a natural environment, and they engage specific psychoevolutionary modes, such as the pleasant affect linked to exploration and approach throughout an environment containing depth and complexity.

There is consistent evidence from early studies that the visual perception of natural environments can improve indices of well-being, such as reduced anxiety, pro-social behavior and reduced physiological symptoms of stress (Berto, 2014). A meta-analysis of 32 studies found that contact with natural environments was associated with a moderate increase positive affect and a smaller, but statistically robust, decrease in negative affect (McMahan and Estes, 2015). Specifically, there is emerging evidence for the proposal that it is the *fractal geometry* of visual scenes of nature that is unique, provides aesthetic pleasure, and may specifically engage this mode of attention (Purcell et al., 2001). In turn, it is proposed that when a person becomes aware of these perceptual qualities of nature, they experience a connectedness to nature that is part of their identity (Heerwagen and Orians, 2002; Schultz, 2002). The Connectedness to Nature Scale (CNS) is a standardized self-report scale to assess this construct (Mayer and Frantz, 2004). It correlates with overall life satisfaction and various facets of well-being (Mayer and Frantz, 2004; Howell et al., 2011; Wolsko and Lindberg, 2013). Consistent with this view, engagement with natural beauty has been found to mediate the relationship between nature connectedness and well-being (Zhang et al., 2014).

Attention restoration theory (ART) suggests that spending time perceiving nature helps to improve focus and concentration levels through replenish an attentional resource required to focus and inhibit distractions (Kaplan and Kaplan, 1989; Kaplan, 1995). Emerging evidence from the beneficial effects of nature on cognitive task performance supports this proposal (Berto, 2014), including a meta-analysis of 42 studies in which improvements in working memory and cognitive flexibility were identified (Stevenson et al., 2018). It is claimed that nature engagement creates these effects by engaging a model of attention known as “fascination” that is effortless, thereby allowing attentional resource to be restored.

A distinct but related approach points to the role of nature engagement in helping to balance the biological systems for managing threat, pursuing reward and seeking comfort to restore homeostasis and allow self-reflection (Richardson, 2019). This approach links nature engagement to the understanding of compassion and mindfulness. For example, there is also emerging evidence that nature connectedness correlates with other factors involving attention–particularly mindfulness, as supported by a recent meta-analysis of 12 studies (Schutte and Malouff, 2018). The relationships vary depending on the facet of mindfulness assessed, and there are indications that *reflective self-attention* may indeed be the closer correlate (Richardson and Sheffield, 2015). The self-regulation approach builds on evidence of this kind, in describing the processes through which many aspects of the self—mastery, attitudes, values—are regulated (Korpela, 1995). Yet a further strand of research goes beyond self-reflection, to assess the role of awareness of a superordinate perspective on the world, akin to mysticism or spirituality (ecological-self theory, Bragg, 1996; transpersonal psychology, Ferrer, 2002; general sense of connectedness, Passmore and Holder, 2017), and there is evidence that this also mediates between connection to nature and well-being (Kamitsis and Francis, 2013; Trigwell et al., 2014). Most recently, drawing upon Gibson's work (e.g., Gibson, 1979), an ecological stance toward nature connection has been described to explain the capacity for nature to enable healthy actions through its wide variety of affordances and niches, such as trees to climb and a wide expanse to explore (Araújo et al., 2019; Brymer et al., 2020). This ecological stance has been encapsulated within a broader humanistic and existential approach to explain, for example, the benefits of extreme sports in natural environments (Immonen et al., 2018). A related conceptualization utilizes attachment theory and phenomenology to describe the way that people's fundamental relationships with nature (e.g., as a secure base; as an embodied interaction) contribute to, and support, the sense of self (Schweitzer et al., 2018).

**Figure 1:**
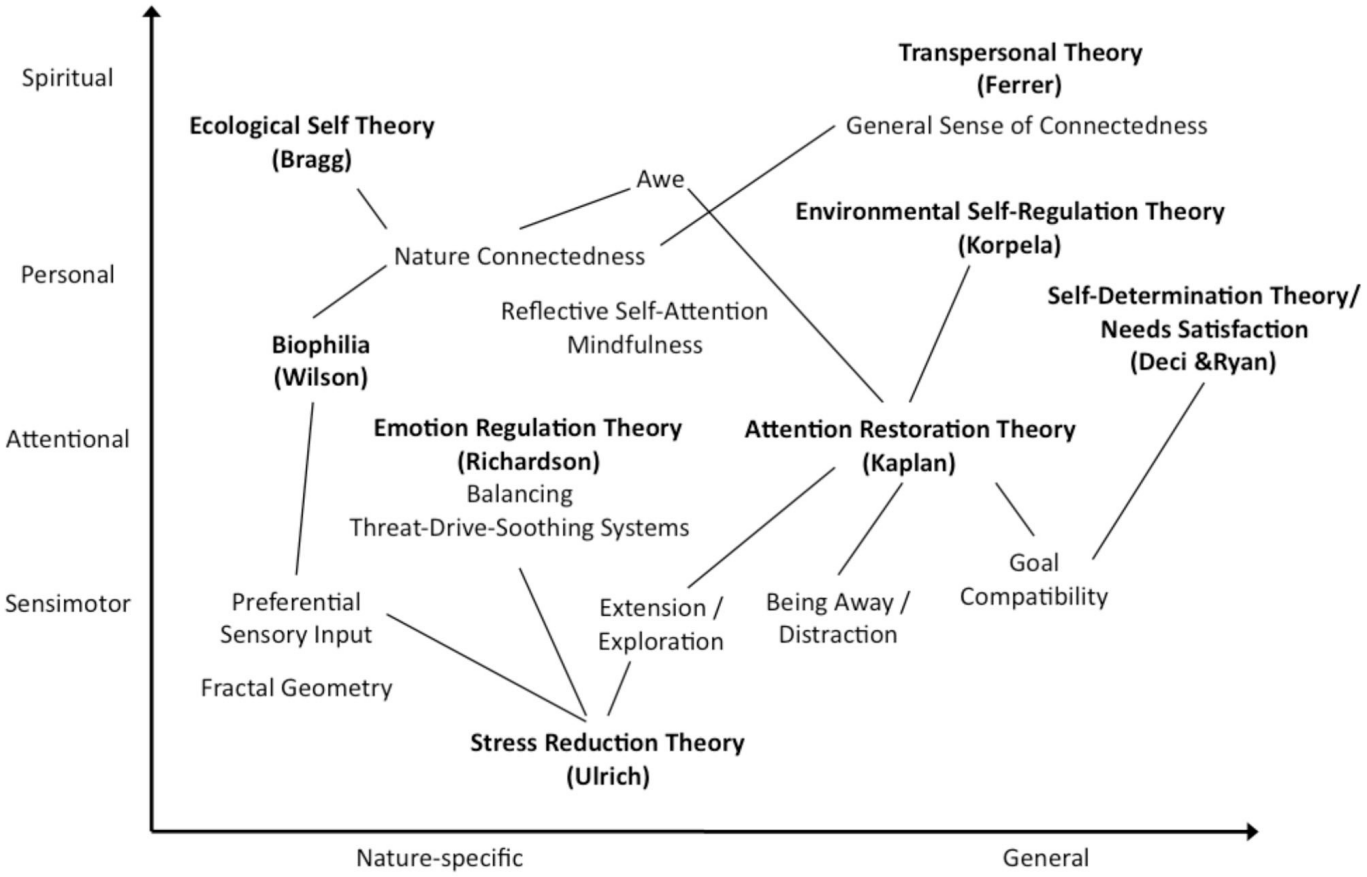
A visualization of the relationship between theories of the well-being benefits of nature engagement. The x-axis represents the degree to which the theory tends to focus on mechanisms that are specific to the biological affinity of humans with natural environments (e.g., evolved biological systems, ecological identity), vs. more general psychological mechanisms that are nonetheless facilitated by nature (e.g., attention, self-regulation), and the y-axis represents the level of abstraction of the components of the theory, from sensimotor processes, through attentional, personal (self-related), and spiritual (transpersonal) components. The ecological-existential/phenomenological-psychoanalytical approach by Brymer et al. encompasses the full spectrum of both axes.

In order to attempt to organize this literature, Figure 1 displays the main theories of nature engagement alongside the main mechanisms of action that they specify. Provisionally, these can be organized in two dimensions to show their approximate relationship. They vary in the level of process that they tend to describe–sensimotor control, attention, self, spiritual—and they vary in terms of the degree to which their mechanisms are specific to nature or more widely applicable. There is however no formal assessment of their overlap, and it is the aim of the current study to focus on specifying mechanisms from the perspective of the individual engaging with nature.

The research literature clearly points to a number of mechanisms. Whilst theories such as ART and ecological-self theory clearly presume a complex inter-relationship between mechanisms at multiple levels—sensory, attentional, self-concept, spiritual—this provides a challenge for quantitative research and statistical modeling. In contrast, this challenge may be addressed by an interdisciplinary framework grounded in biology and engineering, known as perceptual control theory (PCT; Powers et al., 1960a,b; Powers, 1973, 2008). It may be more productive to utilize a theory that is universal and interdisciplinary so that: (a) the biopsychosocial domains of nature engagement can be integrated, and (b) the benefits of nature engagement can be integrated with other interventions such as psychological therapy (e.g., Greenleaf et al., 2014; Jordan, 2014), sports (Barton and Pretty, 2010; Allan et al., 2020), and the arts (Case, 2005).

PCT proposes that organisms control their sensory input through a process that is an extension of homeostasis, mediated externally. Humans, and other organisms, achieve and maintain internal reference values for sensory input at their desired states by acting against disturbances in the environment. These controlled perceptions of the self and the environment are organized hierarchically such that more abstract, long-term experiences are maintained through setting the *reference values* of the next level down. For example, a person who wants to live a worthwhile life may uphold principles regarding their affinity with nature, which entails that they go for regular hikes, during which time they search out and attend to configurations of the natural world that they find calming and pleasurable. Table 1 illustrates 11 levels of perception in PCT and provides examples within nature engagement. Note that in PCT, action is used to control sensory input via “feedback functions” in the environment; this contrasts with the view that affordances in the environment provide sensory input to drive action, as proposed within ecological psychology (Gibson, 1979).

**Table 1:** The perceptual levels within PCT (Powers, 1998) and examples of how they may be involved during nature engagement.

**Name of Level**	**Definition**	**The perceptual levels within one example of nature engagement**
System concepts	Conceptual organizations of principles	Being a nature lover
Principles	Abstracted rules, values and standards	Getting the most of being outdoors
Programs	Nested structures of test and choice points regarding sequences	Tree climbing
Sequences	Experiences that occur in a specific temporal order	Get up the tree; sitting on branch; looking at view
Categories	Classes of perceptions that have shared characteristics	Seeing many different trees
Relationships	The ways in which two or more lower level perceptions relate to one another	Getting on top of a branch
Events	A short, immediate, succession of transitions	A “step” upwards
Transitions	A change in configuration over time, e.g., closing, extending	The bending of the branches
Configurations	A combination of sensations that is perceived as a distinct object	The shape of the branches
Sensations	A vector of intensities	The green color of leaves
Intensities	A signal directly from a sensory organ	The brightness of reflected sunlight from the tree

*This table is organized with the higher levels at the top, but it is best read from the bottom upwards because each higher level perception is formed from the perceptual levels below. The hierarchy is branched rather than linear, but for ease of explanation, just one example is provided at each level*.

PCT proposes that chronic disruptions in well-being can emerge from internal conflict; this is the attempt to make an experience match two or more, incompatible reference values. When this occurs, attention needs to be directed and sustained above the systems in conflict so that an intrinsic, trial-and-error learning process, known as reorganization, can adjust neural strengths and connections until the conflict is resolved. Thus, PCT provides a biologically grounded framework for human decision making, conflict resolution, well-being enhancement and maintenance (Carey et al., 2014); it also provides a framework for computational modeling of psychological pathways (Mansell and Huddy, 2020). Notably, PCT has influenced the development of theories used within the nature engagement field such as choice theory (Glasser, 1981) and wild system theory (Jordan, 2008). PCT also has an evidence base across the disciplines most relevant to nature engagement, including ethology (Pellis and Bell, 2020), neuroscience (Yin, 2017), mental health (Alsawy et al., 2014), and sociology (McClelland, 2020).

Whilst an advanced quantitative research design might model and test potential pathways from nature to well-being specified by PCT in the future, an empirical bridge can be provided by qualitative research. Within open self-reports, people who perceive improvements in well-being from engaging with nature can, within the limits of introspection, provide multiple themes regarding the mechanisms they notice as important.

Examples of the most relevant qualitative research studies that have explored the pathways from nature engagement to well-being are summarized in Table 2. There are many more examples of studies that have focused on specific activities, such as various hobbies and sports, which are not summarized here.

**Table 2:** Examples of earlier qualitative studies and their findings regarding the pathways between nature engagement and well-being.

**References**	**Methods**	**Key findings**
Windhorst and Williams (2015)	12 students prompted to photograph the elements of their natural environment that they felt contributed to their well-being and mental health	Participants chose environments that were familiar, an escape, and enabled self-reflection
Martyn and Brymer (2016)	305 students. Analysis of text-based answers to the question of “what being in nature means to you”	Seven themes: sensory engagement, expanse, connection (including social and nature connectedness), time out, relaxation, enjoyment (including feelings of restoration), and a healthy perspective
McCree et al. (2018)	11 disadvantaged children given highly interactive, child-centered interviews	A meta-theme of “establishing self-regulation and resilience through emotional space.” Additional themes of autonomy, freedom, meeting basic needs, engaging in physically adventurous activity, free social play, discovering about nature, becoming socially confident learners, making choices, and gaining independence
Coventry et al. (2019)	45 adults engaging in conservation activities in public green spaces	Degree of personal meaningfulness and control over the specific activity related to a sense of satisfaction
Birch et al. (2020)	24 young multi-ethnic urban residents	Three themes of relational felt sense within nature were described: self-acceptance, escape, and connection and care
Brymer et al. (2021)	15 people who reported enhanced well-being from leisure activities in nature	Three overarching themes were “a sense of perspective,” “mental and emotional sanctuary,” and “being immersed in the moment,” supporting an interactive relationship between people and the natural environment

The qualitative studies to date have echoed the findings within the quantitative research, but this small number of studies has also expanded the scope of mechanisms at multiple levels of experience (sensory, attentional, self-concept, etc), and across intrapersonal, interpersonal and systemic (e.g., school) domains. The most prominent strand running through them appeared to be how natural environments provide a spectrum of opportunities for people of all ages to meet their multiple biological and psychological needs: independence/autonomy/control, physical activity, emotional expression and problem resolution, play, discovery, personal growth, self-reflection, self-acceptance, and social connectedness. Given the limited number of studies, but their wide scope and limited integration to date, we designed an interview study that aimed to identify and organize the potential pathways within an interdisciplinary framework.

In order to meet the above aim, we required a methodology that met the following criteria: (1) a sample who identified that nature engagement activities had benefited their well-being and/or mental health and were therefore motivated to talk in detail about potential mechanisms; (2) a convenient sample that were articulate, available to devote the time, and able to reflect on the benefits that nature had during their lives—therefore undergraduate university students were recruited; (3) a semi-structured interview that focused on identifying potential pathways that emerged from the participants themselves, rather than specific questions regarding specific pathways. We aimed to compare our findings with the wider empirical literature to interpret them within the context of theories of the benefits of nature engagement and the nature of subjective, social and psychological well-being. In order to do so, we adopted an inductive approach, guided by the idiosyncratic experiences of the clients. Following this we examined existing gaps in theory and explored whether PCT might help integrate the pathways within an analytical model, specific to the findings of the current study.

## Methods

### Design

This study used qualitative research methods, through a semi-structured interview.

### Participants

The study was advertised through an email from the university communication email service, requesting for undergraduate students who have experiences with nature engagement in their childhood and adolescence, and who are willing to talk about the role of experience with nature in their mental health and well-being. Table 3 shows the participants demographics and what nature-related activities they engaged in.

**Table 3:** Participants' characteristics.

**Pseudonyms**	**Age range**	**Gender**	**Ethnicity**	**Nature-related activities engaged with**
Marian	18–25	F	Italian/Romanian	Climbing, walking dogs, pet therapy, playing with animals
Polly	18–25	F	White British	Hiking, scouts (hiking, camping, fires, learning about nature), gardening, tree planting, walks, outdoor yoga, bug hunts, painting/art, canoeing, swimming, feeding animals, cleaning animals
Liz	40–50	F	White British	Walking in the countryside, farming/harvesting, caring for animals, swimming in the sea, playing on the beach, surfing
Anne	18–25	F	Belgian	Hiking, walking, running, climbing
Harper	18–25	F	White British	Forest walks, playing in the park, looking for birds, animal engagement, feeding animals
Nina	18–25	F	Indian	Walking, nature-based craft activities
Aimen	18–25	F	Pakistani	Gardening, walking, playing with sand, farming

### Materials

The topic guide was developed by the first author, reviewed and discussed with the second author, and piloted with two individuals reporting benefits of nature engagement before being refined as the study version (Table 4), The interviews were recorded using an encrypted audio recorder.

**Table 4:** Topic guide for the semi-structured interview questions.

Please could you start by telling me about the nature-related activities you liked as a child. Which of these activities do you still engage in?
What was it about these activities that you liked?
How would you say that these activities impacted your well-being or mental health as a child? Why do you think it impacted you in this way? Does it still affect you?
Can you describe an example of when these nature-related activities benefited your well-being? How do you think it helped?
If you were not able to engage in these activities, how do you think it would affect your well-being? Was there a time when you weren't able to do them? How did it make you feel? Why?
Do you think that any of these activities has affected you as an individual? now? If so, could you explain to me how you think it has had this effect on you?
Is there anything else you would like to cover that I may have missed?
Do have any question you would like to ask?

### Procedure

After establishing email contact, the participants were emailed an information sheet about the study, explaining the aims of the study and what was required of them. The interviews took place in a testing cubicle in the Psychology department of the university. When the participant arrived at the interview, they were asked to complete a consent form and a short demographic form. The semi-structured interviews was conducted, the participants were thanked for taking part in the study and they had the opportunity to ask any questions.

All the interviews were transcribed. After the recordings were fully transcribed, each audio recording was deleted from the encrypted file. The first author carried out a thematic analysis using Braun and Clarke's guidelines (Braun and Clarke, 2006). Thematic analysis is a technique to categorize themes and patterns across a dataset about the research question (Braun and Clarke, 2013). Thematic analysis is flexible, allowing the researcher to answer a research question with ease. It uses a bottom-up approach to identify themes. The first step the first author took in generating themes was by familiarizing and understanding the data set. The first author read the transcripts multiple times to get to know the data set and to think if there are any common answers or patterns thought all the interviews. Then the first author started to code the transcripts, which allowed her to develop an understanding of the data set further. The first author then grouped the common codes into meaningful categories, narrowing the choices for themes. The next step was to make potential themes, which resulted in 11 themes. Finally, the second author reviewed the themes and their associated quotes. After discussion, it was decided that the previous names did not accurately capture the essence of each theme and so these were updated and finalized.

## Results and Discussion

After applying thematic analysis to the transcriptions, 11 themes emerged from the data set. These will be explained in a hierarchical order from sensory experiences upwards, similar to the vertical dimension of Table 1 and Figure 1, culminating in the themes that illustrate the apparent overall function of nature engagement in participants' lives. Each theme is discussed in relation to the wider theory and evidence, and a complementary, integrative role of PCT is introduced.

### Theme 1: Enjoy the Different Sensory Inputs

This theme is about how the participants enjoyed the sensory input they received from nature, such as visual, auditory, tactile, and olfactory. The sensory input gave the participants a pleasing sensory experience. The participants explained how they highly appreciated different sensory contributions. Some participants explained that they valued a broad horizon and the different colors from the natural environment, other valued the quietness and the sounds that came from natural sources such as trees, or the smell of nature, or even the feeling of grass. Polly said:

“When I go home, I just feel so much better and as much as I enjoy being at uni and it's a whole new different lease of life, going home, seeing the stars again because there's no light pollution, lying on the ground feeling the grass.”

This demonstrated that the participants liked the simple effects that nature gave them, possibly due to the minimal effort for mental processing. It was easy for them to take in the different sensory experiences; it does not involve feelings of stress or anxiety. In the natural environment, there are no proper requirements. Therefore, it enabled individuals to take in the different modes of sensory input. It could be that an individual's subconscious allowed them to experience how peaceful and quiet nature is. Harper said:

“I'd say on a concrete level, literally the fresh air, like when I breathe in, I think it tastes different… the feeling the fresh air and seeing and I feel like there are a lot of colors are going on with it and you get the smells of flowers and plants and its quite sensory.”

Participants described how nature allowed them to explore the scene, and there were diverse experiences they could get from their environment. As Harper explained, the sensory input is on a concrete level. Therefore, the eyes, ears, nose, and hands have to a specific degree, evidence, that their senses were fulfilled with what they subconsciously enjoy. Participant reported the natural sounds such as rain, leaves rustling, and the sea that had a calming effect, and described how that this effect brought joy. Similarly, with the other sensory inputs, they had other positive effects, for e.g., smelling flowers and freshly cut grass, or looking at a vast horizon was aesthetically pleasing to the eye, which in turn led to participants' reports of enjoyment.

The increase in positive affect and the reduction of unpleasant affect encapsulate a facet of subjective well-being (Diener et al., 1999), and the role of sensory input from nature in affect regulation nature has been a key theme of earlier research (e.g., Ulrich, 1979; Martyn and Brymer, 2016). Indeed, the theme of “immersion in the present moment” in a recent qualitative study described the role of the richness of perceptual experience within a natural environment that facilitates absorption (Brymer et al., 2021). Moreover, the engagement with natural beauty has been found to mediate the relationship between nature connectedness and happiness (Richardson and McEwan, 2018). As mentioned in the Introduction, the biophilia hypothesis explains these sensory preferences for nature as evolutionary in origin (Wilson, 1984), their facilitation of evolved affective systems are described in SRT (Ulrich, 1983) and these are operationalised as goal-directed sensory the preferences within ART (Kaplan, 1995). These theories make the fair assumption that humans have sensory preferences, and PCT has the grounding within engineering and mathematics to describe the working mechanism in detail. According to PCT, the behavior of humans and other animals is the manifestation of controlled sensory input and this can be modeled as a negative feedback control system (Powers, 1973). Taken together, our participants' expression of the enjoyment of sensory input from nature may provide neurophysiological balance precisely because it permits them to act, freely, to control the input to their senses as they observe and navigate through a natural environment.

### Theme 2: Feel Freedom

This theme is about how nature-related activities made people feel as if they were free and liberated, often because they did not need to conform with the expectations of others. Marian said:

“You can get to be yourself because no one is judging you and is looking at you, like animals don't care about the way you look or whatever you're doing, they're just there with you. And you don't have to force it.”

The participants described feeling free to roam around and explore, and they seemed to link this to some of the other benefits of nature. For example, Nina said:

“I would feel a lot more free and you can separate yourself from the things that are stressing you and allows to problem solve.”

The autonomy facet of psychological well-being (Ryff and Keyes, 1995) comes closest to the feeling of freedom theme described here, and it has emerged in other qualitative studies (e.g., Martyn and Brymer, 2016; McCree et al., 2018). Given the subjective nature of this theme, it is hard to assess objectively, yet there is a small but robust benefit in attentional control—the ability to orient and sustain attention of one's own volition—measured after nature engagement (Stevenson et al., 2018). The feeling of freedom is implicit within theoretical accounts of nature engagement, such as the goal compatibility feature of ART describes the benefits of experiences that are self-selected, also a component of self-regulation theory (Korpela, 1995) and self-determination theory (Deci and Ryan, 1985). PCT echoes this self-regulatory function; the intrinsic need to control one's sensory experiences through freedom of action is at the heart of PCT, guiding psychological development throughout the lifespan (Plooij, 2020). Method of Levels, a therapy based on PCT, provides clients with the freedom to control their own access to therapy and the choice of problem to discuss, and the therapist uses questioning to facilitate the client's ability to shift and sustain attention to various facets of the experiences they choose to discuss (Carey, 2006; Mansell, 2018).

### Theme 3: Calm Nature Facilitates a Calm Mood

Participants explained that nature is calm in terms of sounds and the overall atmosphere; it influenced the individual's feelings. The atmosphere corresponded to their emotions. The participants described various specific examples of how experiences of nature enabled their calm and relaxed mood. Marian said:

“It can change your mood, definitely. Like the calmness of the trees helps you to be calm as well.”

Similarly, Nina said:

“The feeling of breeze and fresh air on your skin and the sounds in nature are softer so trees rustling and animals and those more gentle sounds. So they are very calming for me. And also the feeling of grass, laying on the grass.”

In each case, there was a confluence between the gentle nature of the experiences of nature and the calming, relaxed mood the participants experienced.

The feeling of calmness and relaxation form a component of subjective well-being (Diener et al., 1999). The capacity for the aesthetic qualities of natural scenes to faciliate calm mood forms one of the affective reactions described in SRT (Ulrich, 1983). More recently, Richardson's emotion regulation theory describes a neurophysiological pathway for this effect in substantial detail, via the balancing of the parasympathetic (PNS) and sympathetic (SNS) nervous systems (Richardson, 2019) and it builds upon research indicating that the majority of individuals experience an increase in PNS and a decrease in SNS activation when engaged with nature [reviewed by Richardson (2019)]. There is convergent evidence that the PNS sustains a reflective mindset that in itself is an aid to well-being via its impact on self-reflection, perspective-taking and decision-making (Kok and Fredrickson, 2010); this links the current theme with additional themes extracted in the study, as discussed later. Yet, it is important to recognize that individuals in certain circumstances experience a perceived danger within natural environments, and this may be experienced as undesirable (e.g., animal phobias) or even as desirable (e.g., in outdoor risk-taking pursuits; Lupton and Tulloch, 2002). Thus, the potential for nature engagement to be calming may be one, albeit important, function regarding emotion regulation (Richardson, 2019). The role of “calming” mood within a PCT framework is described as it emerges in later themes.

### Theme 4: Enhancing Decision Making and Forming Action Plans

This theme refers to how nature-related activities allowed individuals to make a concrete action plan and make decisions. The participants explained how they found it easier to make plans when they engaged with nature relative to when they were inside. Anne said:

“I'm working and I'm inside, all these different thoughts pop up and I can get a bit disorganized and then when I'm outside I think it's much easier to organize my thoughts a little bit more and come up with an action plan. And then really come back inside and get back to what I need to do… it allows me to see the different things that I need to do and organize them.”

Engaging with nature helped the participants to clear their mind. It helped to stop thinking about unnecessary thoughts, that was previously on the participant's mind, which led to more excessive unhelpful thinking patterns, and enabled them to contemplate on the essential factors. For example, Liz said:

“A platform to solve problems… if I take myself out of a situation and I can look at it more objectively and that's what nature gives to me. To overcome the panic that instilled and with that clarity, you can start to put plans in place to work with the challenge.”

This theme clearly reflects the pathway to well-being described within ART and in particular the importance of “being away” to escape from distraction, reduce urgency, and engage a more reflective mode of thought (Kaplan, 1995). Decision-making and action planning are mental processes that are also integral to self-regulation (Korpela, 1995). The benefit of natural environments for reflective processing have been identified in earlier qualitative studies (e.g., Windhorst and Williams, 2015). Indeed recent years have seen an emergence of studies finding improvement in sustained attention (Pasanen T. et al., 2018) and creative problem-solving after periods of immersion in nature (Atchley et al., 2012; Ferraro, 2015) and there is evidence from structural equation models that the attentional focus facilitated by the natural environment contributes to these benefits (Pasanen T. P. et al., 2018). Our participants did not specifically describe creativity as a benefit of nature engagement, but they did describe the capacity to contemplate their priorities and solve problems. On the face of it, the social, subjective and psychological perspectives on well-being do not describe the capacity to make decisions and action plans as a facet of well-being. Nonetheless, they might be expected to contribute to autonomy and personal growth (Ryff and Keyes, 1995). PCT may complement the above approaches by specifying in more detail the state required for decision-making; it is a neurophysiological state that allows allows conflicted goals to be held in awareness to explore the potential decisions for long enough, and in sufficient detail, to come to a solution through reorganization (Mansell, 2020).

### Theme 5: Enhancement of Efficiency and Productivity

Participants explained that after they engaged with nature-related activities, they were much more productive and efficient in their work, relative to before engaging with the activity. Nina said:

“I am much more productive if I take out some time to go out.”

Participants described engaging with nature as a positive distraction from working because when the individual was working, they focused on one thing for a significant amount of time such as an essay, thus when the individual engaged with nature they had many different things they could divert their attention to for example the sky, the horizon, or an animal. This made the individual feel revitalized, which enabled them to become productive later. Aimen said:

“During exams you say you're going to revise from this time to this time, so you need a break, best thing you can do it that break, is to go spend some time with the natural environment, in nature, with fresh air. It freshens your mind, it gives you more energy, it motivates you, like okay I should go back to what I was doing.”

Participants reported that when they studied but could not focus, and they decided to engage with nature, after the activity, they noticed that their efficiency and productivity levels had increased, allowing them to study more than before they had engaged with nature. Some used it as an effective strategy to use for a break; they can exert some energy, feeling refreshed, as Aimen said “it freshens your mind.”

This theme appears to represent an overarching benefit from the enhanced decision-making and restoration provided by nature, and maps onto aspects of subjective well-being such as the pleasant feeling of energy and refreshment, and work satisfaction (Diener et al., 1999). To some degree, it appeared to also feed into the sense of environmental mastery and autonomy, facets of psychological well-being (Ryff and Keyes, 1995), that participants experienced. Maybe because this theme represents the tail-end of the pathway from nature to well-being, it is not captured as a component of nature engagement theories, but it would be consistent as an outcome of them, especially ART (Kaplan, 1995) and self-regulation theory (Korpela, 1995). There is emerging evidence, building the pathway from creative problem-solving, that dispositional nature engagement is associated with a more innovative cognitive style, which is regarded as a benefit for educational and commercial organizations (Leong et al., 2014). From the perspective of PCT, “productivity” and “efficiency” represent principles within a perceptual hierarchy. Immediately above the level of principles are system concepts, including concepts of the self, others, organizations and the wider world. Thus, to the extent that an individual pursues and upholds the principles of efficiency and productivity within their self-concept, they will experience benefits in well-being from the properties of nature engagement that have these effects.

### Theme 6: Alleviating Pressure From Society's Expectations Regarding Education

Many participants explained how nature-related activities helped them through the difficult education system. The demands of education appeared to be profound in the case of some participants, but the amount of stress varied depending on the individual. Liz said:

“I'm in the cohort of the first people to do GCSE's…the pressure on us was extraordinary…so that 2 years, that 14–16 years was very stressful and I identified a walk that I would go on… because of having that degree of connection, reduced my stress… in a way it continues now, I think at that point things felt like they were going very fast and in nature things don't go fast and they go very slow…. So, it was certainly that morning walk that set me on course to maintain my mental health during school day, it sort of gave me that reflection and preparation time.”

The participants described how the positive effects described within earlier themes helped to lessen the pressure of education. When the individual stepped out of a place that was causing them anxiety and which created a great deal of pressure, nature enabled them not to become overwhelmed. Nina said:

“I was doing my GCSE exams, so when I was about 15, the I would go take breaks at the weekend and go outside for a long walk and because it was a stressful time and I felt very confined when I was inside surrounded by work and they were the biggest exams I had done at that stage in my life, going outside would calm me down and I would feel a lot more free and you can separate yourself from the things that are stressing you and allows to problem solve.”

When the participants got the opportunity to go out, they felt as if they could do things at their time, there was a sense of ease and control. This contrasted with how they described the education system, where control is taken away from students because they are told what to so and when to do it.

This theme may have been specific to the current sample because they were young adults reflecting on their life, which was largely dominated by academic work. Within the domain of employment there is emerging evidence that nature engagement breaks reduce job stress (de Bloom et al., 2017) and there is also evidence that nature engagement in schools leads to improved engagement with classroom education in primary school children (Kuo et al., 2018). The theme involved participants experiencing a break from the pressure of societal expectations regarding education, and the experience of a lack of control over such requirements. This theme therefore points to the importance of understanding multiple levels of experience—including the system concept of society's expectations—which an individual can choose to conform to, or not, at any moment in time. Thus, the facets of psychological well-being such as autonomy and purpose in life may benefit via this pathway. The theme also dovetails with the focus of PCT of the importance of control in well-being, and the necessity to shift awareness to higher levels of perception to achieve change through a shift in perspective. This links the current theme to later themes of both feeling freedom and nature putting life into perspective.

### Theme 7: Formation of Community Relations

This theme is about how individuals formed community relationships through nature engagement activities. The activities were a way toward a strong bond with one another. Polly said:

“Cause I've got a real community there it is obvious that like it's a supplement for my well-being so if I don't have time for it then it doesn't help with the stressful situations at all… scouting is just, has completely shaped my life, the skills that you get there and the people that you meet.”

Not only were these activities a way to build relationships, but these relationships helped to deal with stressful life events that occurred. These types of relationships last a very long time because there is a strong foundation of how the relationship formed, through the mutual enjoyment of a particular activity. Marian said:

“We were out with all the people, we didn't spend time inside, like playing video games or watching television, we literally had to go outside… we kind of like created a team.”

Participants reported that interacting with others improved their mood, and they helped to form tight-knit communities because they could relate with each other through a shared interest.

This theme describes an element of social well-being, especially the sense of connectedness and social support (Larson, 1993). It is also a facet of subjective well-being as satisfaction with one's social group (Diener et al., 1999), and psychological well-being as positive relations (Ryff and Keyes, 1995). The community relations pathway has been noted as a theme in earlier qualitative research (O'Brien et al., 2010; Genter et al., 2015), yet theories of nature engagement have tended to focus on connectedness with nature itself (Bragg, 1996) and spiritual connectedness (Ferrer, 2002), and their interrelationship (Kamitsis and Francis, 2013). Nonetheless, there is evidence that even a 2-week nature-based intervention can promote pro-social orientation and connectedness to other people (Passmore and Holder, 2017), thus potentially improving social well-being and helping to draw upon others as a source of support. From the perspective of PCT, communities are system concepts—the highest level of perception in PCT—and so being part of a community may be fundamental to well-being for many people.

### Theme 8: Nature Puts Things Into Perspective

This theme is about how nature-related activities allowed the participants to improve their viewpoint on a negative situation and enabled them to realize the severity of a situation and how significant, or insignificant, it truly was. Through the change in thought process, they felt better after engaging with nature-related activities. Liz said:

“It's about putting things into perspective quality it seems to have…a period when myself and my other half were looking for a house and we were spending most weekends driving around houses, so we weren't doing our normal leisure stuff at the weekend and moving houses is stressful anyway and I felt my enjoyment in it going down and my frustration in it going up…let's go to the peak district instead and using it again to get things into perspective, get them to a manageable state…I'd sort of thing well the trees don't care; the river doesn't care. It would help me retain perspective, having that calm beginning to the day.”

Engaging with nature allowed the individual to have a connection, by appreciating how amazing nature is and how everything is meant to be in life, and a link was made by putting the negative situation into perspective. For some, it enabled the individual to believe that things would fall into place, giving a sense of hope. Marian said:

“I mean you feel like your problems are so small compared to like how big the forest is like the view from the mountain and everything.”

A comparison was made with nature to the problem, realizing that it was not as significant as they previously thought. The participants tended to treat the problem as an animate object when comparing it to the natural environment. For example, Marian said “your problems are so small compared to like how big the forest is.” This comparison was then used to help the individual understand that they could manage their hardships.

This theme appeared to illustrate how nature could help people re-establish their purpose in life, a facet of psychological well-being (Ryff and Keyes, 1995). The capacity for nature to provide a beneficial higher level perspective on one's life struggles is a feature of a number of theories, including the spirituality of transpersonal theory (Ferrer, 2002), the perspective of an ecological self (Bragg, 1996), and the role of the awe experienced through witnessing natural environments as described within ART (Kaplan, 1995). This pathway was elucidated by a recent qualitative study of leisure engagement in nature that described the theme of “a sense of perspective” to include a sense of oneness with nature, and feelings of humility and gratitude in the face of the awe of nature. The perspective provided by nature also receives support from studies indicating that nature connectedness mediates the benefits of nature (Kamitsis and Francis, 2013; Trigwell et al., 2014). Interestingly, the sustained awareness of a higher level perspective on one's conflicting goals at any level, is the mechanism of recovery from the distress experienced by chronic goal conflict as described by PCT (Powers, 1973); thus the perspective-widening capacity of nature may be another fundamental pathway to well-being.

### Theme 9: Liking the Contrast From the Urban Environment

Participants made a clear contrast between the natural environment and the urban environment. They reported that the urban environment can be hectic, where an individual needs to watch out for cars, bikes and general traffic, whereas when in nature there is no need to be as cautious because such things are not an issue. Liz said:

“Leaving behind man-made noise and entering an environment where there is only natural noise has a distinct neurological effect on you. It seems to calm you and make you more alert at the same time.”

The participants reported that the natural environment brings about a sense of peacefulness, possibly because there is not much pressure on cognitive functioning; for example, Liz said “seems to calm you.” Participants reported that in the urban environment, people have to pay attention to their surroundings to look out for danger, even for simple tasks such as changing buses in the city center. Harper said:

“I feel like when you live in the city, when the cars zoom past it subconsciously causes a bit of anxiety in you, whereas in nature the calmness and stillness, like the contrast between the city and natural environment, you feel less anxious subconsciously because of the difference.”

The contrasting features of the urban and natural environment are integral to the many theories of nature benefits and implicit within qualitative studies. The reasons for this contrast relates to the various nature engagement pathways (e.g., the contrasting sensory input). Nonetheless, we extracted this theme because the participants overtly stated and elaborated upon the contrast. Indeed there is evidence within research on self-regulation theory that people hold “place identities” for natural locations—places where they feel an emotional bond or strong sense of attachment (Ratcliffe and Korpela, 2018) and these may be the natural locations that they realize meet their psychological needs (Landon et al., 2020). Within PCT, this choice of current surroundings would involve programs (e.g., routines, plans such as hikes, fishing trips, forest school, etc) that are situated in the middle of a goal hierarchy between the higher levels of principles and system concepts, and the lower levels that control more immediate experiences such as the choice of landscapes, plants and animals to attend to. Controlling programs of this kind would enable them to experience the desired qualities of natural environment over the urban environment.

### Theme 10: Coping Mechanism

Many of the participants explicitly used nature as a coping mechanism. If they were stressed out or going through a difficult time, they engaged in nature-related activities, which helped them cope with their emotion. Polly said:

“I just have to go in nature to feel a bit better. So I can literally go lie on the ground and look at the sky and even though it won't solve the problem, it will temporarily make me feel a bit better, so I can go back practically deal with the situation.”

This theme drew upon the earlier themes of sensory input as enjoyable, and nature as calming, because the process of coping drew upon these qualities of nature-related activities. For example, participants described how they would seek out nature when stressed and it would help them to relax. For example, in some cases, attention was diverted to something aesthetically pleasing such as stars in the sky; for example, Polly said “go in nature to feel a bit better.” Therefore, being around a pleasing aspect of nature enabled the individual to place their attention to something enjoyable to look at, thus resulted in positive psychological changes in the body. Anne said:

“I get really stressed, so I'll go outside and go for a walk for a few minutes… So being able to go outside and clear my head really helps… It's both a coping mechanism and also preventative because I know that if I don't do it influence me.”

Nature engagement as coping is a theme within earlier studies (e.g., Chawla, 2020). Coping has some overlap with the facets of psychological well-being such as autonomy and the concept of environmental mastery (Ryff and Keyes, 1995). It is also a key process within self-regulatory theories of nature engagement (Korpela, 1995), and “coping” as a construct may link together the fields of ART, emotion regulation and self-regulation, in that the choice to engage with nature to cope with stress may enable restore the balance of the PNS and SNS systems, and through its impact on attentional processes, also restore concentration (Berto, 2014). PCT may be able to integrate the themes described earlier to specify a specific pathway for long-term coping–the reduction of chronic unresolved goal conflict (Powers, 1973). It pinpoints the reason for a misbalance in physiological systems as chronic suppression of “action-ready” bodily systems that are thwarted by conflicting goals (Mansell, 2020). Therefore, long-term coping emerges when people can select the kinds of environments that allow them to engage with and reflect upon their goal conflicts and resolve them through reorganization. Reduction in arousal and improvement in concentration then may emerge as the conflict is reduced. It is possible that this property of natural environments leads them to be considered by some as a “mental and emotional sanctuary” (Brymer et al., 2021).

### Theme 11: Anxious if Prevented or Restricted

Participants explained how if the participants were prevented from engaging in nature-related activities, they would become anxious. Liz said:

“I'd notice anxiety going up, yeah. And it could creep up on you, if you spend a couple of months and for some reason you've not managed to do it and haven't managed to get out of town, I just find myself more worried about everything.”

The participants were aware that because their nature engagement activities were used as a stress release, when their means to release stress was taken away, anxiety increased. Harper said:

“I feel like I would get antsy…I would be more stressed out and like anxious and I just wouldn't be enjoying it.”

It is a logical conclusion that anxiety and stress may resurface when access to nature is limited, given its benefits for stress reduction. Nonetheless, the participants' were particularly aware of this dependence on nature in their accounts and its effect on their subjective well-being (Diener et al., 1999). Their experience could be interpreted as a validation of the view that access to nature is a basic need owing to our biological heritage (Wilson, 1984). Alternatively, it could be claimed that using a “dose” of nature to “cope” is insufficient, in a similar way to the resurgence of anxiety during withdrawal from medication for emotional disorders. A potential integrative stance is that nature engagement has a unique and intrinsic capacity to combine multiple pathways to well-being, yet it is not always sufficient in facilitating benefits that sustain. Future research could explore whether certain forms of nature engagement, possibly those that facilitate higher-level perspectives on conflicting aspects of the self, as described in PCT, have long-term benefits.

## General Discussion

**Figure 2:**
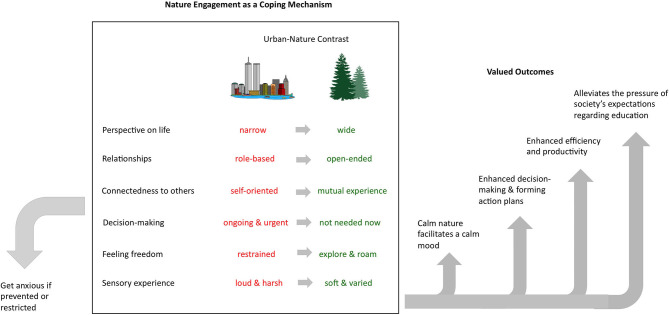
A diagram of the themes in the study, organized to illustrate the multi-level pathways through which nature engagement as a coping mechanism leads to multi-level benefits to well-being. The themes outside the box are worded as in the Results and Discussion. However, within the box, the rest of the themes have been organized hierarchically, and the Urban vs. Nature theme has been used to contrast each of these perceptions between the two environments using material from participants' accounts.

The combination of themes in the current study is novel, and also somewhat comprehensive, seeming to encompass many of the themes from earlier qualitative studies, many of which also have an empirical basis within quantitative research. Therefore, we attempt to fully integrate them here (see Figure 2).

The bottom line inside the box in Figure 2 describes the lower levels of perceptual experiences within the natural environment. These levels of perception would include intensities, sensations, configurations and transitions within the PCT hierarchy. The specific aesthetic qualities of these perceptions within the natural environment and how they facilitate adaptive neurophysiological modes of action are well-described by SRT, ART and Emotion Regulation Theory. Key examples of their beneficial pathways emerging from the analysis are a calm mood (a valued outcome in Figure 2), and a free exploration of experience (going up a level in Figure 2), which in turn enables creative problem-solving (a further valued outcome in Figure 2) which in turn leads to further valued outcomes including improved efficiency and productivity (Figure 2). Interestingly, the perceptual hierarchy in PCT can short-circuit the exterm environment and enter an imagination mode whereby stored perceptions are experienced “as if” real to the brain (Powers, 1973). This capacity has been used to explain the role of imagery in psychological interventions (e.g., Mansell and Hodson, 2009), and there is evidence that mental imagery of natural scenes may be particularly beneficial, for example in reducing state anxiety (Nguyen and Brymer, 2018).

As well as providing a hierarchical framework, PCT describes the nature of a “problem” and how it is solved. A person is primed to act on a goal, entailing physiological arousal, yet becomes stuck with these feelings, which are now experienced as stress owing to thwarted goal progress, and this requires a creative solution (Mansell, 2020). PCT proposes that the solution will emerge if attention is directed and sustained above the systems in conflict until the process of reorganization allows spontaneous, trial-and-error changes that resolve it. It is notable therefore that there are higher levels of perception in Figure 2—connection with a community and a wider view on one's life from the perspective of one's connection with the natural world. In order to resolve issues relating to one's own conflicting standards or social roles, PCT would indicate that a person needs to shift and sustain their awareness above these levels to eventually resolve them, and one of these resolutions includes alleviation from the pressures of the education system (the final valued outcome in Figure 2). The additional themes in Figure 2 are outside the box because they indicate that, owing to the above benefits, natural environments themselves are a controlled perception, to be used as a coping strategy (a “program” in PCT terms), and anxiety is experienced when this control of environment is restricted or not possible.

Importantly, this model is analytical rather than formal, and the aim of future research will be to formalize these components within a PCT architecture and construct individualized models that can be tested for their fit with real world data within natural environments. This methodology is challenging, but it provides a particularly robust test of a psychological theory (Mansell and Huddy, 2020). The functional specification of a model will also facilitate the research and translational impact within new technology, such as smartphones and augmented or virtual reality, which is a burgeoning area in nature engagement research (Wooller et al., 2018; MacIntyre et al., 2020; Yin et al., 2020). Arguably, the closest conceptualization to date to encompass the multi-layered benefits of nature engagement is the phenomenological-psychoanalytic, ecological-existential framework of Brymer and colleagues (e.g., Immonen et al., 2018; Schweitzer et al., 2018). One clear distinction is that perceptual control theory provides a single theoretical framework, in contrast to a pluralistic integration of multiple theories.

There were a number of limitations of this study. First, although the sample were of the desired age range and showed some ethnic diversity, only university-educated female participants were recruited. Therefore, different themes may have emerged with a larger sample of a wider range of education and a representative balance of genders. Second, the semi-structured interview format was retrospective. This was an advantage in that the research captured a historic overview of the most personally meaningful aspects of nature. However, it relied on memory and focused on general themes rather than idiosyncratic, dynamic experiences. In future, using distributed ethnographic models with participants self-coding would increase the granularity of the findings and provide mode detail on the context of the findings for future research.

Overall, this study has replicated and extended the themes from earlier qualitative studies exploring the pathways of benefit from nature engagement. These themes have been parsed into a multiple levels and analyzed in the context of constructs of well-being, a range of theories of nature engagement benefits, and perceptual control theory to generate an integrative account to guide future research and practice.

## Data Availability Statement

The raw data supporting the conclusions of this article will be made available by the authors, without undue reservation.

## Ethics Statement

The studies involving human participants were reviewed and approved by UREC, University of Manchester. The patients/participants provided their written informed consent to participate in this study.

## Author Contributions

WM conceived of the study and co-designed it. AI ran the study and the data analysis. Both authors wrote the manuscript.

## Conflict of Interest

The authors declare that the research was conducted in the absence of any commercial or financial relationships that could be construed as a potential conflict of interest.
